# Infants born during COVID-19 pandemic experience increased susceptibility to airway hyperresponsiveness

**DOI:** 10.3389/falgy.2024.1512182

**Published:** 2024-12-16

**Authors:** Idit Lachover-Roth, Anat Cohen-Engler, Yael Furman, Yossi Rosman, Keren Meir-Shafrir, Michal Mozer-Mandel, Sivan Farladansky-Gershnabel, Tal Biron-Shental, Ronit Confino-Cohen

**Affiliations:** ^1^ Allergy and Clinical Immunology Unit, Meir Medical Center, Kfar Saba, Israel; ^2^ Faculty of Medical and Health Sciences, School of Medicine, Tel Aviv University, Tel Aviv, Israel; ^3^ Department of Obstetrics and Gynecology, Meir Medical Center, Kfar Saba, Israel

**Keywords:** allergy, atopic comorbidities, COVID-19, hygiene hypothesis, airway hyper responsiveness

## Abstract

**Background:**

Asthma, allergic rhinitis, atopic dermatitis, and food allergy are type 2 inflammation diseases. Since the 1960s, the prevalence of those diseases has steadily increased, presumably due to the “Hygiene hypothesis” which suggests that early exposure of infants to pathogens, siblings, and environmental dust, has a protective effect against the development of allergic diseases. The COVID-19 pandemic increased environmental hygiene due to lockdowns, masks, and social distancing.

**Objective:**

To compare the prevalence of allergic diseases among children born before and during the pandemic.

**Methods:**

The Cow's Milk Early Exposure Trial prospectively followed newborns until 12-months of age using monthly survey and examined milk allergy development. Some were born before the first COVID-19 lockdown in Israel (April 2018–March 2020), and some were born during the pandemic (March 2020–May 2021). The monthly surveys included questions regarding atopic comorbidities.

**Results:**

A total of 1,989 infants completed 12-months of follow-up. Among them, 1,086(54.5%) were diagnosed with at least one atopic disease. Among 235 infants born after the last lockdown, 162 were diagnosed with airway hyperresponsiveness (AHR)(68.9%), significantly more than in any other group. No other significant differences were found between the study groups.

**Conclusions:**

There was no significant difference in the development of atopic comorbidities between infants born before and during the pandemic. Significantly more infants who were born after restrictions were eased were diagnosed AHR. A longer follow-up period is needed to obtain a better understanding of the influence of the COVID-19 restrictions on the development of atopic comorbidities.

**Clinical Trial Registry:**

NIH Clinical Trials Registry: NCT02785679.

## Introduction

Atopic dermatitis (AD), food allergy (FA), asthma, and allergic rhinitis (AR) are grouped under the term “atopic diseases” (1). These diseases have a common immunological mechanism based on T helper 2 (Th2) cells and therefore also termed “Type 2 inflammation diseases” (2). Usually, the first atopic disease that develops in young infants is AD, during the first months of life (1). Later, FA and asthma [termed airway hyperresponsiveness (AHR) develop in infants and toddlers]. Finally, allergic rhinitis usually develops at the end of the first or the beginning of the second decade of life (3). However, other studies suggest several patterns of timing (4).

Since the 1960s, the prevalence of all atopic diseases has steadily increased (5). According to the Israeli Ministry of Health, 25%–40% of the Israel population has at least one atopic disease (6). The National Survey of Children's Health reported that the prevalence of children with at least one atopic disease in the United States is 35.1% (7). The main explanation for this surge is partially based on the “Hygiene Hypothesis” and it is new version, the “Old Friends” Hypothesis (8–10). Those theories suggest that early exposure to allergens, foreign substances, and pathogens has a protective effect against the development of allergic diseases, as it preserves the balance between T helper 1 (Th1) and Th2 cells (5). Children in the Western world are exposed less to pathogens and more exposed to antibiotics, both of which are suggested as a cause for the increased prevalence of atopic diseases and impaired balance between Th1 and Th2 (11). This theory is supported by data showing that children who were exposed at a young age to other children, such as siblings or in day-care, were less likely to develop atopic diseases later in life (12, 13). Other immune cells are involved in the development of type 2 inflammation or its suppression including T regulatory and T helper 17 cells (14). Although many observational studies have provided evidence that support the “Hygiene hypothesis”, this hypothesis has not been examined prospectively (8, 12, 13).

On March 11, 2020, the World Health Organization announced the Coronavirus disease 2019 (COVID-19) as a world pandemic (15). The COVID-19 pandemic increased environmental hygiene, due to lockdowns, mask use, and social distancing. From March 2020–May 2021, Israel had three lockdowns, the population was required to wear masks, and kindergartens were closed for a total of more than 100 days (16). During this period, the prevalence of common infectious diseases, such as influenza and other non-COVID infections decreased significantly (17, 18).

Based on the hygiene and old friend hypotheses together with these changes in daily practices, it is reasonable to postulate that in the years to come, the prevalence of atopic diseases among children who were born during the COVID-19 pandemic will be higher compared to those who were born before it.

The study hypothesis is that atopic comorbidities will be more frequent in infants who were born during the COVID-19 pandemic.

## Methods

This observational prospective longitudinal study is based on 2,252 infants recruited to the Cows' Milk Early Exposure Trial (COMEET) from April 2018 until May 2021 at a single center in Israel (19). Briefly, infants were recruited shortly before labor and evaluated monthly by phone or e-mail survey until 12 months of age. Addition details were published previously (19). The infants continue to be followed annually until the age of ten years (ongoing study). As this study started two years before the pandemic manifested in Israel and continued to recruit infants during the COVID-19 pandemic, it offers an exceptional opportunity to compare the prevalence of atopic diseases in the first year of life between infants born before, during, and after the pandemic. The current study focused only on the first 12 months of life.

The COVID-19 outbreak in Israel was first noticed on March 11, 2020, and social distancing was recommended (20). On March 19, 2020 the government announced the first lockdown (21). Overall, Israel had three lockdowns (March–May 2020, September–October 2020, and December 2020–February 2021).

The study cohort was divided into two groups, each with 2 subgroups:

- Group A: Pre-pandemic group (included all infants born from April 2018–March 10_,_ 2020).
  ○ Sub-group A1: Not affected by the pandemic during the first 12 months of life (infants older than 12 months on March 10, 2020).
  ○ Sub-group A2: Pre-pandemic group, affected by lockdowns (infants 0–12 months of age on March 10, 2020).
- Group B: Pandemic group (all infants born from March 11_,_ 2020, until the end of recruitment in May 2021).
  ○ Sub-group B1: Lockdowns group (infants born during the lockdowns, March 10, 2020, to February 7, 2021, the end of the third lockdown).
  ○ Sub-group B2: Post-lockdowns group (infants born after lockdowns, since February 8, 2021, until the end of recruitment in May 2021).

### Study variables

The preliminary, monthly, and yearly questionnaires included information on family history of atopic diseases, perinatal data (gestational age at birth, birth weight, mode of delivery, medical events during the perinatal period, infectious diseases, antibiotic treatment, and vaccinations).

Evaluation of atopic comorbidities (i.e., AD, FA, and AHR) was based on parental reports regarding physician diagnosis, treatments provided, and tests performed as previously described (19). AHR is defined if the infant needs inhalations with *β* agonist and corticosteroid (ICS).

REDCap software was used for data collection.

The original study and its extension were approved by the local ethic committee. Participants' parents provided signed informed consent to participate in the study.

### Statistical analyses

Only infants completing 12 months of follow-up were included in the data analysis. The characteristics of the study groups were compared using the Chi-squared test (binary and categorical variables). *P*-values were reported without correction for multiplicities. Sub-group analyses were conducted according to use of antibiotics, mode of delivery, number of siblings, nursing setup at 12 months of age, and the original groups included in the COMEET study. A hierarchical, binary logistic regression was performed to determine the relationship between AHR; study groups (A1, A2, B1, and B2); sex, ethnic group, mode of delivery, nursing setup, vaccination, siblings, and family atopic background, at the categorical level. All statistical analyses were performed using SPSS-27 (IBM Corp., Armonk, NY, USA).

## Results

**Figure 1:**
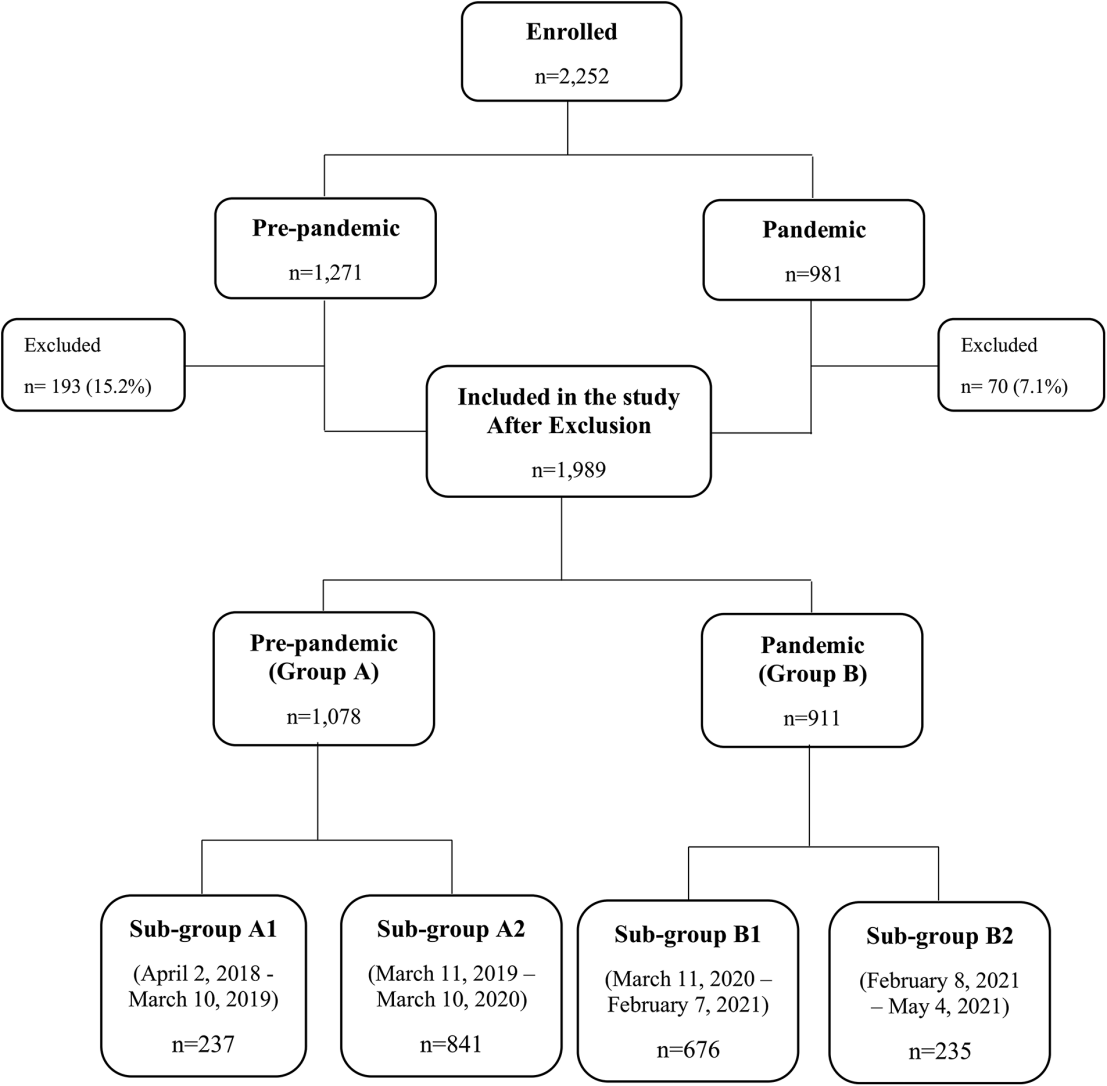
Distribution of the study groups. Study groups were formed according to the pandemic periods and are not equal. Excluded infants: 263 infants were excluded because they did not complete 12 months of follow-up: 100 were lost to follow-up, 158 consent withdrawn, and 5 developed serious medical problems. Details are presented in Supplementary Table S1.

During April 2018 and May 2021, 2,252 infants were recruited to the COMEET study. Among them, 1,989 (88.3%) infants completed 12 months of follow-up. Data analyzed in the current study included 1,078 (54.2%) infants born before the pandemic (pre-pandemic—Group A), and 911 (45.8%) born during the pandemic (pandemic—Group B). Within group A, 237 infants (22%) were older than 12 months at the beginning of the pandemic (Sub-group A1), and 841 (78%) were 0–12 months old (Sub-group A2). Within group B, 676 (74.2%) were born during lockdowns (lockdowns sub-group—Sub-group B1), and 235 (25.8%) were born after the lockdowns (post-lockdowns sub-group—Sub-group B2). Figure 1 presents the distribution of the study groups. The demographic characteristics of the groups are detailed in Table 1. The number of infants born each month is presented in Supplementary Figure 1.

**Table 1:** Demographic characteristics of the study groups.

Variables			Pre-pandemic (Group A)		Pandemic (Group B)		Total (*n* = 1,989)	*P*-value*
			Sub-group A1 (*n* = 237)	Sub-group A2 (*n* = 841)	Sub-group B1 (*n* = 676)	Sub-group B2 (*n* = 235)		
Sex	Female		124 (52.3%)	425 (50.5%)	326 (48.2%)	119 (50.9%)	997 (50.1%)	NS
Socioeconomic status (mean)			7	7	7	7	7	NS
Ethnic group	Jewish		217 (91.6%)	743 (88.3%)	607 (89.8%)	204 (86.8%)	1,771 (89%)	NS
	Arab		20 (8.4%)	98 (11.7%)	69 (10.2%)	31 (13.2%)	218 (11%)	NS
Mode of delivery	Vaginal		**207 (87.3%)^a^**	748 (89%)	620 (91.7%)	**222 (94.5%)^b^**	1,798 (90.4%)	**0.043**
	Cesarean Section	Total	30 (12.7%)	93 (11%)	56 (8.3%)	13 (5.5%)	192 (9.6%)	NS
		Elective	14 (5.9%)	39 (4.6%)	17 (2.5%)	4 (1.7%)	74 (3.7%)	NS
		Emergency	16 (6.8%)	54 (6.4%)	39 (5.8%)	9 (3.8%)	118 (5.9%)	NS
COMEET study groups	Exclusive breastfeeding		122 (51.5%)	461 (54.8%)	368 (54.4%)	120 (51.1%)	1,071 (53.8%)	NS
	Breastfeeding + cow's milk formula feeding		67 (28.3%)	265 (31.5%)	202 (29.9%)	82 (34.9%)	616 (31%)	NS
	Cow's milk formula feeding only		48 (20.3%)	115 (13.7%)	106 (15.7%)	33 (14%)	302 (15.2%)	NS
Nursing setup at 12 months of age	Home with parent		**48 (20.3%)^a^**	**298 (35.5%)^b^**	**203 (30%)^b,c^**	**54 (23%)^c^**	603 (30.3%)	**<0.05**
	Private caregiver		19 (8%)	53 (6.3%)	49 (7.2%)	22 (9.4%)	143 (7.2%)	NS
	Daycare		**170 (71.7%)^a^**	**488 (58.2%)^b^**	424 (62.7%)	159 (67.7%)	1,241 (62.5%)	**<0.05**
Vaccines during the first year			227 (95.8%)	**779 (93.2%)^a^**	**656 (97%)^b^**	**231 (98.3%)^b^**	1,893 (95.4%)	**<0.01**
Number of siblings	No siblings		90 (38%)	289 (34.4%)	198 (29.3%)	73 (31.1%)	650 (32.7%)	**<0.05**
	Siblings	Total	147 (62%)	553 (65.7%)	478 (70.7%)	162 (68.2%)	1,339 (67.3%)	**<0.05**
		1	70 (29.5%)	281 (33.4%)	224 (33.1)	83 (35.3%)	659 (33.1%)	NS
		2	59 (24.9%)	198 (23.5%)	171 (25.3%)	55 (23.4%	483 (24.2%)	NS
		3 or more	18 (7.6%)	73 (8.7%)	83 (12.3%)	24 (10.2%)	198 (9.9%)	**<0.05**
Family atopic comorbidities**	Parental		55 (23.2%)	229 (27.2%)	201 (29.7%)	64 (27.2%)	549 (27.6%)	NS
	Mother		36 (15.2%)	146 (17.4%)	137 (20.3%)	47 (20%)	366 (18.4%)	NS
	Father		24 (10.1%)	127 (15.1%)	88 (13%)	28 (11.9%)	267 (13.4%)	NS
	Siblings		33 (13.9%)	163 (19.4%)	132 (19.5%)	39 (16.6%)	367 (18.4%)	NS

Group A: Pre-pandemic group (infants born from April 2018 until March 10_,_ 2020. Sub-group A1: Not affected by the pandemic (infants over 12 months of age on March 10, 2020). Sub-group A2: Pre-pandemic, affected by lockdowns (infants 0–12 months old on March 10, 2020).

Group B: Pandemic group (infants born from March 11, 2020, until May 2021). Sub-group B1: Lockdowns group (infants born during the lockdowns). Sub-group B2: Post-lockdowns group (infants born after lockdowns).

NS, not significant; COMEET, cow's milk early exposure trial.

* *P* value refers to differences between all sub-groups A1, A2, B1, and B2. Superscripts a, b, c, and d indicate between which groups the difference was significant (all “a” in the same line are similar, and significantly differ from b, c, or d. accordingly, all “b” in the same line is similar). The significant differences are highlighted in bold.

** Family atopic comorbidities include at least one of the following: asthma, atopic dermatitis, food allergy, and allergic rhinitis.

In infants born during the pandemic period (Group B), vaginal delivery was much more common than caesarean section (C/S) compared to the pre-pandemic group (A) (842/911 92.4% vs. 956/1,078 88.6%, *p* = 0.004). They also had more siblings than the pre-pandemic group did (640/911 70.3% vs. 699/1078 64.8%, *p* = 0.013).

Infants in sub-group A1 were at daycare more than infants in sub-groups A2 and B1 who stayed at home with a parent (71.7% vs. 58.2% vs. 62.7%, respectively, *p* < 0.05), and did not exhibit significant differences compared to infants in sub-group B2 (post-lockdowns) (71.7% vs. 67.7% respectively, *p* = 0.368). Infants in sub-group A2, pre-pandemic, affected by lockdowns, had significantly fewer routine vaccines than infants in sub-groups B1 and B2 (93.2% vs. 97% and 98.3%, respectively, *p* < 0.001; Table 1).

### Infants' morbidities during the first year of life

Infants in sub-group B2, post- lockdowns group, were more frequently diagnosed with AHR than infants in sub-groups A1, A2, and B1 [147/235 (62.6%) vs. 106/237 (44.7%) vs. 341/841 (40.5%) vs. 319/676 (47.2%), respectively, *p* < 0.001; Table 2]. The significant difference in the incidence of AHR is represented by the use of β agonist and ICS inhalations. There were no significant differences in the prevalence of AD and FA between the groups or subgroups (Table 2).

**Table 2:** Infants' morbidities during the first year of life.

Clinical characteristics		Pre-pandemic (Group A)		Pandemic (Group B)		Total (*n* = 1,989)	*P*-value*
		Sub-group A1 (*n* = 237)	Sub-group A2 (*n* = 841)	Sub-group B1 (*n* = 676)	Sub-group B2 (*n* = 235)		
Courses of antibiotics	0	**109 (46%)^c^**	**695 (83.5%)^d^**	**371 (54.9%)^c^**	**133 (56.6%)^c^**	1,308 (66.1%)	**<0.01**
	1	89 (37.5%)	97 (11.7%)	159 (23.5%)	46 (19.6%)	391 (19.8%)	**<0.001**
	2	**18 (7.6%)^c^**	**27 (3.2%)^c^**	**91 (13.5%)^d^**	**29 (12.3%)^d^**	165 (8.3%)	**<0.001**
	3 or more	21 (8.9%)	**13 (1.6%)^c^**	52 (7.7%)	**27 (1.1%)^d^**	113 (5.7%)	**<0.05**
Atopic comorbidities	Total	**123 (51.9%)^a^**	**429 (51%)^a^**	**372 (55%)^a^**	**162 (68.9%)^b^**	1,086 (54.5%)	**<0.01**
	Atopic dermatitis	34 (14.3%)	127 (15.1%)	100 (14.8%)	34 (14.5%)	295 (14.8%)	NS
	Airway hyperresponsiveness	**106 (44.7%)^a^**	**341 (40.5%)^a^**	**319 (47.2%)^a^**	**147 (62.6%)^b^**	913 (45.8%)	**<0.001**
	Food allergy	5 (2.1%)	22 (2.6%)	12 (1.8%)	4 (1.7%)	43 (2.2%)	NS

Group A: Pre-pandemic group. Sub-group A1: Not affected by the pandemic (infants over 12 months of age on March 10, 2020). Sub-group A2: Pre-pandemic, affected by lockdowns (infants under 12 months old on March 10, 2020).

Group B: Pandemic group (infants born from March 11, 2020, until May 2021). Sub-group B1: Lockdowns group (infants born during lockdowns). Sub-group B2: Post-lockdowns group (infants born after lockdowns).

* *P* value refers to differences between Groups A & B, and between all sub-groups A1, A2, B1, and B2. Superscripts a, b, c, and d indicate between which groups the difference was significant (all “a” in the same line are similar, and significantly differ from b, c, or d. accordingly, all “b” in the same line is similar). The significant differences are highlighted in bold.

As the major contributor to the differences between the prevalence of atopic comorbidities was the prevalence of AHR, in the rest of the results, we refer only to this difference.

The differences between sub-group B2 and the other sub-groups remained significant after adjusting for possible confounders (Table 3). The prevalence of AHR was also influenced by the following confounders: C/S delivery, staying at home with parents, and fed with cow's milk formula only. The difference remains significant in multivariate analysis after adjustment for the confounders (*p* < 0.001), detailed in Supplementary Table 2.

**Table 3:** The influence of demographic and optional clinical confounders on the prevalence of hyper-reactive airway disease.

Confounders		Odds Ratio between sub-groups (95% CI)			
		A_1_ vs. B_2_	A_2_ vs. B_1_	A_2_ vs. B_2_	B_1_ vs. B_2_
Antibiotic use	No	0.549 (0.327–0.922)*	0.945 (0.73–1.223)	0.611 (0.421–0.887)*	0.646 (0.434–0.963)*
	Yes	0.302 (0.168–.543)**	0.85 (0.567–1.274)	0.305 (0.171–0.543)**	0.358 (0.212–0.605)**
Mode of delivery	Vaginal	0.469 (0.318–0.690)**	0.746 (0.602–0.923)**	0.397 (0.291–0.54)**	0.532 (0.388–0.728)**
	C/S	0.75 (0.203–2.766)	0.963 (0.492–1.883)	0.619 (0.193–1.986)	0.643 (0.191–2.16)
Siblings	No	0.492 (0.263–0.921)*	0.927 (0.641–1.341)	0.474 (0.282–0.798)**	0.511 (0.297–0.88)*
	Yes	0.484 (0.335–0.7)**	0.763 (0.622–0.936)*	0.408 (0.303–0.55)**	0.535 (0.395–0.725)**
Nursing setup at 12 months of age	Home	0.526 (0.263–1.05)	0.833 (0.59–1.174)	0.552 (0.333–0.915)**	0.633 (0.394–1.116)
	Day-care	0.417 (0.264–0.658)**	0.757 (0.583–0.983)*	0.369 (0.251–0.543)**	0.487 (0.329–0.721)**
Feeding during the first two months	Exclusive breastfeeding	0.374 (0.222–0.629)**	0.708 (0.534–0.938)*	0.283 (0.186–0.432)**	0.401 (0.261–0.615)**
	Breastfeeding (exclusive or in combination with cow's milk formula)	0.472 (0.315–0.708)**	0.778 (0.624–0.972)*	0.397 (0.288–0.547)**	0.509 (0.367–0.708)**
	Only cow's milk formula feeding	0.526 (0.212–1.303)	0.707 (0.416–1.202)	0.488 (0.22–1.085)	0.69 (0.308–1.546)

Sub-group A1: Not affected by the pandemic (infants over 12 months of age on March 10, 2020). Sub-group A2: Pre-pandemic, affected by lockdowns (infants 0–12 months old on March 10, 2020). Sub-group B1: Lockdowns group (infants born during the lockdowns). Sub-group B2: Post-lockdowns group (infants born after lockdowns).

Differences between sub-group A1 vs. A2, and A1 vs. B1 were not significant in all the parameters examined, and therefore data not shown.

NS, not significant; C/S, Cesarian section.

* *p* < 0.05.

** *p* < 0.01.

Regarding antibiotic treatment: Infants in sub-group A2 received fewer antibiotics treatment than all the other sub-groups (Table 2). Overall, the prevalence of AHR in infants, from all the sub-groups, who were treated with antibiotics was higher than among those who were not during their first year of life [391/671 (58.3%) vs. 519/1,308 (39.7%), respectively; *p* < 0.001]. Looking at them as two separate groups, infants who received antibiotics in group B, were diagnosed with AHR significantly more often than were group A infants who received antibiotics [139/265 (52.5%) vs. 252/406 (62.1%), respectively, *p* = 0.014]. There was no significant difference in the diagnosis of AHR between infants in groups A and B (and sub-groups) who were not treated with antibiotics during their first year of life [305/804 (37.9%) vs. 214/504 (42.5%), respectively; *p* = 0.104].

### Use of inhalations during the first year of life

**Figure 2:**
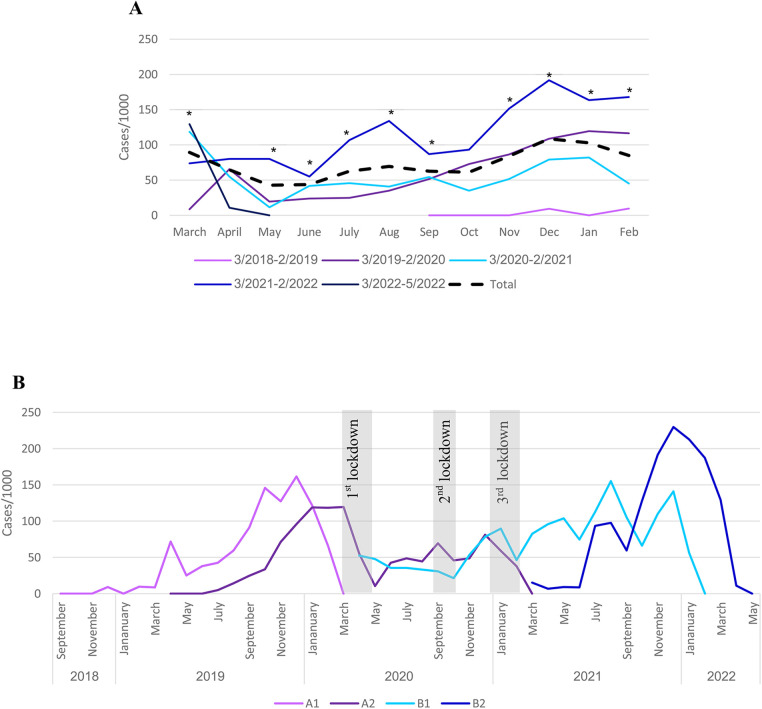
Seasonal inhalation uses during the study. **(A)** Inhalation use distribution along the year. Light purple line includes sub-group A1. The dark purple line includes sub-groups A1 and A2. Light-blue line includes sub-groups A2 and B1. The blue line includes sub-groups B1 and B2, and the dark blue line includes sub-group B2. *p < 0.001 March 2021-February 2022 (blue line) and March 2019-February 2020 (dark purple line). **(B)** Inhalation use in the sub-groups. The Gray bars represent the lockdowns in Israel. Each line represents a separate sub-group.

Infants required more inhalations during the winter season (November–March). This trend remained stable throughout the study period, April 2018–May 2022 (Figure 2A), except for July–August 2021. During the first year of the COVID-19 pandemic, March 2020–February 2021, when Israel had over 100 days of lockdowns (16), the rate of inhalation use was lower compared to the previous and following years (Figure 2A). The trend during the next year, March 2021–February 2022, that was based only on infants born during the pandemic restrictions, was much higher compared to the average and previous years. The differences between the period March 2021–February 2022 to that of March 2019–February 2020, were statistically significant throughout the year, except for April and October (*p* < 0.001).

When analyzing the data for each sub-group separately (Figure 2B), the difference between the years is mainly due to infants belonging to the post-lockdowns sub-group B2, that had the highest use.

## Discussion

The “hygiene hypothesis” and its new version, the “Old Friend” hypothesis are currently the leading theories used to explain the constant increase in the prevalence of atopic comorbidities. The COVID-19 pandemic gave the medical world a unique opportunity to examine those hypotheses prospectively and objectively. The current study prospectively followed newborns from the first day of life until the age of 12 months. There was no significant difference in the development of AD and FA between infants born before and during the pandemic. Infants that were born after the pandemic restrictions were eased (from February 2021), were diagnosed significantly more with AHR. Although AHR is not strictly a type 2 inflammation disease, it is well established that infants suffer from AHR as infants and toddlers are more prone to develop asthma as school-age children (22, 23).

The COMEET study (19) served as an ideal platform for prospective evaluation of those hypotheses, as about half of the participants were enrolled before the COVID-19 pandemic (before March 2020), others during its eruption and associated restrictions, and a group born after most restrictions were removed. As far as we know, this is the largest prospective cohort that examines the influence of the COVID-19 pandemic on the development of atopic comorbidities. Other studies included fewer than 600 infants (24, 25).

The current study did not find that an “over-hygienic” environment caused an increase in the prevalence of allergic comorbidities during the first year of life. On the contrary, we found that infants born in the “post-lockdowns” period (sub-group B2) even though they did not receive more courses of antibiotics, they required significantly more β-agonist inhalations and ICS than the study groups born before or during the pandemic restrictions. There could be several reasons for this observation. Infants in sub-group B2 tend to visit daycare facilities more frequently than those born just before and during the pandemic-related restrictions (sub-groups A2 and B1). However, this alone does not explain why they experience a higher incidence of AHR compared to infants in sub-group A1, who were not directly influenced by the pandemic and had a similar daycare attendance pattern to sub-group B2. This discrepancy may be attributed to the increased prevalence of respiratory infections observed after the easing of restrictions (26). In contrast to the Israeli Center of Disease Control annual report (27) and a large study from China (28), Amar et al. found that Israeli children ages 0–3 years experienced an unexpected increase in the incidence of non-COVID-19 respiratory infections in the months after the restrictions were eased, beginning in April 2021 (26). Furthermore, Dagan et al. have documented a surge in the incidence of RSV, Adenovirus, and human metapneumovirus beginning in April 2021 (29). Those observations support our findings, and the high rate of AHR in sub-group B2. Another explanation for the increase in inhalation use in the post-lockdowns sub-group could be an increase in the severity of respiratory syncytial virus (RSV) infections that were observed after COVID-19 restrictions were eased (30). One speculation for the heightened severity observed could be attributed to the fact that the mothers were likely not exposed to RSV during pregnancy, possibly due to its absence from circulation and the COVID-19 restrictions. Consequently, the transfer of RSV antibodies to their fetus, which typically occurs, might not happened (31–33). The relationship between RSV bronchiolitis and recurrent wheeze is known and can explain the incidence of inhalations use in sub-group B2 (infants born at the post-lockdowns period). However, it is not known what is the cause and what is the result—does infants who are prone to recurrent wheeze develop more severe RSV bronchiolitis or infants who have severe RSV bronchiolitis are more prone to develop recurrent wheeze (34).

The low incidence of AHR found in sub-groups A2 and B1 (infants affected by the lockdowns) is probably because of the low incidence of respiratory infections during the pandemic restrictions (18). This is in concordance to the results of a small retrospective study that compared the prevalence of respiratory morbidity during the first year of life between infants born at the beginning of the pandemic (February–March 2020) and a matched group of infants born during the same period in 2019 (24). That study reported that infants born at the beginning of the pandemic had significantly fewer respiratory comorbidities (24). The same results were shown in the CORAL birth cohort (25, 35) that compared infants born in Ireland during the first two months of the pandemic to a United Kingdom birth cohort from 2008 as a control group. The first year of life of the infants in those two studies was during the pandemic when there were fewer respiratory and influenza infections (18), similar to sub-groups A2 and B1.

In contrast to what we expected to find based on the hygiene and old friend hypotheses, we did not find an increase in the prevalence of atopic comorbidities in infants born before and during the pandemic in our study groups. Possible explanations for this observation include: A. The pandemic changed not only the hygiene level, but also other parameters including fewer Cesarean sections and more infants staying at home with their parents for the first 12 months of life. Moreover, environmental changes, including the diversity of pathogens were noted (36) and even air pollution (37) was dramatically reduced during lockdowns. Do those changes affect the trend and prevalence of atopic comorbidities? Maybe the following years and a longer follow-up period might provide this information. B. Although all four atopic comorbidities are grouped, they are different diseases with different mechanisms of acquisition. We speculate that asthma (presented until the age of 5 years as AHR) and AR are more sensitive to viral exposure and respiratory infections. A longer follow-up period is needed to find significant differences in these two diseases.

As far as our knowledge extends, this study marks the pioneering attempt to investigate the influence of the COVID-19 pandemic and its associated restrictions on the development of atopic comorbidities. While the elevated incidence of AHR may serve as an initial indicator of the pandemic's effect on the prevalence of other atopic comorbidities, it is imperative to conduct further investigations before drawing broad-reaching conclusions.

We are planning to follow the COMEET study cohort until the age of 10 years. We anticipate that longer follow-up will provide a better understanding of the influence of the COVID-19 restrictions on the development of atopic comorbidities.

This study had several limitations: First, the groups were not randomized, and several characteristics differed significantly between the groups. We stratified the groups to control these differences. Second, the COMEET study was designed for a different purpose and the questionnaires were designed accordingly. This led to some deficits in the data, including COVID-19 infections that might have influenced the results. Third, the infants in group B2 were born in a very short period and seasons (the end of winter and part of the spring), and this differs from the other groups that were born in all seasons. However, because we have a representation of all seasons we can compare the trends between the different groups. Additionally, Infants in sub-group A2 received fewer routine vaccinations without changes in the National immunization schedule. We assume this difference is technical due to lockdowns and restrictions that made it difficult to schedule visits for routine vaccines. However, this did not influence the prevalence of AHR.

This study is the largest prospective study in infants analyzing the incidence of atopic comorbidities during a real-life situation of hygiene restrictions.

## Conclusion

Infants born at the end of the COVID-19 pandemic restrictions (after February 2020) had more AHR and received more inhalations than infants born before and during the pandemic restrictions. We did not find significant differences in the prevalence of atopic comorbidities between infants born before, during or after the pandemic lockdowns. The results of this study suggest that the hygiene hypothesis is probably only one of many factors involved in the complex pathogenesis of atopy. However, a longer follow-up might provide different results.

## Data Availability

The raw data supporting the conclusions of this article will be made available by the authors, without undue reservation.
