# Supplementary material for: Infants born during COVID-19 pandemic experience increased susceptibility to airway hyperresponsiveness
Source: Front Allergy. 2024 Dec 16;5:1512182. doi: 10.3389/falgy.2024.1512182 (PMC11683114; doi:10.3389/falgy.2024.1512182)
Supplement: Supplementary file 1 [file Table1.docx]

**Supplement Figure 1**: Number of infants recruited according to month of birth.

Excluded infants are not included in the graph.

**Supplement Table 1.** Reasons for exclusion according to study group

| **Reason** | **Group A1** | **Group A2** | **Group B1** | **Group B2** | **Total** |
| --- | --- | --- | --- | --- | --- |
| **Lost to follow-up** | 12 (13.9%) | 54 (50.5%) | 27 (49.1%) | 7 (46.7%) | 100 (38%) |
| **Revoked consent** | 73 (84.9%) | 51 (47.7%) | 27 (49.1%) | 7 (46.7%) | 158 (60.1%) |
| **Serious health problem** | 1 (1.2%) | 2 (1.9%) | 1 (1.8%) | 1 (6.6%) | 5 (1.9%) |
| **Total** | 86 (32.7%) | 107 (40.7%) | 55 (20.9%) | 15 (5.7%) | 263 |

Group A1: Not affected by the pandemic (infants who were over 12 months of age on March 10, 2020). Group A2: Pre-pandemic, affected by lockdowns (infants who were 0-12 months old on March 10, 2020). Group B1: Lockdowns group (infants born during the lockdowns, March 10, 2020, to February 7, 2021, the end of the third lockdown). Group B2: Post-lockdowns group (infants born after lockdowns, since February 8, 2021, until the end of recruitment in May 2021).

**Supplement Table 2.** Logistic regression model comparing the prevalence of airway hyperresponsiveness between the study groups

| **Group** | | **B** | **Odds Ratio** | **95% CI** | | **p value** |
| --- | --- | --- | --- | --- | --- | --- |
|  |  |  |  | Lower | Upper |  |
| **Constant** | | -1.244 | 0.288 |  |  | <0.001 |
| **Pre-pandemic (A)** | **A_1_** |  | 1 |  |  |  |
|  | **A_2_** | -0.95 | 0.909 | 0.672 | 1.229 | 0.536 |
| **Pandemic (B)** | **B_1_** | 0.127 | 1.136 | 0.835 | 1.545 | 0.418 |
|  | **B_2_** | 0.765 | **2.150** | 1.470 | 3.145 | <0.001 |
|  | | Nagelkerke pseudo r^2^ = 0.094, χ^2^ =3.462 | | | | |

Model adjusted for sex, ethnic group, mode of delivery, original study group at the COMEET study, vaccination, nursing setup at 12 months, siblings, and family atopic background.

Group A1: Not affected by the pandemic (infants who were over 12 months of age on March 10, 2020). Group A2: Pre-pandemic, affected by lockdowns (infants who were 0-12 months old on March 10, 2020). Group B1: Lockdowns group (infants born during the lockdowns, March 10, 2020, to February 7, 2021, the end of the third lockdown). Group B2: Post-lockdowns group (infants born after lockdowns, since February 8, 2021, until the end of recruitment in May 2021).
